# Postoperative delirium after cardiac surgery of elderly patients as an independent risk factor for prolonged length of stay in intensive care unit and in hospital

**DOI:** 10.1007/s40520-021-01842-x

**Published:** 2021-04-03

**Authors:** Andrea Kirfel, Jan Menzenbach, Vera Guttenthaler, Johanna Feggeler, Andreas Mayr, Mark Coburn, Maria Wittmann

**Affiliations:** 1 Department of Anaesthesiology and Intensive Care Medicine, University Hospital Bonn, Venusberg-Campus 1, 53127 Bonn, Germany; 2 Institute for Medical Biometry, Informatics and Epidemiology, University Hospital Bonn, Venusberg-Campus 1, 53127 Bonn, Germany

**Keywords:** Postoperative delirium, Cardiac surgery, Elderly patients, Diagnosis of delirium, Length of stay

## Abstract

**Background:**

Postoperative delirium (POD) is a relevant and underdiagnosed complication after cardiac surgery that is associated with increased intensive care unit (ICU) and hospital length of stay (LOS). The aim of this subgroup study was to compare the frequency of tested POD versus the coded International Statistical Classification of Diseases and Related Health Problems (ICD) diagnosis of POD and to evaluate the influence of POD on LOS in ICU and hospital.

**Methods:**

254 elective cardiac surgery patients (mean age, 70.5 ± 6.4 years) at the University Hospital Bonn between September 2018 and October 2019 were evaluated. The endpoint tested POD was considered positive, if one of the tests Confusion Assessment Method for ICU (CAM-ICU) or Confusion Assessment Method (CAM), 4 'A's Test (4AT) or Delirium Observation Scale (DOS) was positive on one day.

**Results:**

POD occurred in 127 patients (50.0%). LOS in ICU and hospital were significantly different based on presence (ICU 165.0 ± 362.7 h; Hospital 26.5 ± 26.1 days) or absence (ICU 64.5 ± 79.4 h; Hospital 14.6 ± 6.7 days) of POD (*p* < 0.001). The multiple linear regression showed POD as an independent predictor for a prolonged LOS in ICU (48%; 95%CI 31–67%) and in hospital (64%; 95%CI 27–110%) (*p* < 0.001). The frequency of POD in the study participants that was coded with the ICD F05.0 and F05.8 by hospital staff was considerably lower than tests revealed by the study personnel.

**Conclusion:**

Approximately 50% of elderly patients who underwent cardiac surgery developed POD, which is associated with an increased ICU and hospital LOS. Furthermore, POD is highly underdiagnosed in clinical routine.

## Introduction

Postoperative delirium (POD) is an adverse and underdiagnosed postoperative complication of elderly patients [1–3]. Data on the incidence of POD in surgical populations varies in a broad range from 11 to 51% [2]. Defined by the Diagnostic and Statistical Manual of Mental Disorders, Fifth Edition (DSM-5) and the 10th revision of the International Statistical Classification of Diseases and Related Health Problems (ICD-10), delirium is an acute and fluctuating disturbance of awareness, attention and cognition caused by an organic pathophysiology [4, 5]. In the literature, POD is divided into different forms, hyperactive, hypoactive and a mixture of both. Especially, the hypoactive delirium often remains undetected in the average clinical setting because of its characteristics such as unawareness, decreased alertness and decreased motor activity [6–10].

Numerous risk factors are associated with the development of POD [11]. In addition to predisposing factors of the patient such as age, comorbidities, cognitive and functional impairment, the treatment of the patient like surgical invasiveness and duration of the operation are also causative for POD [2]. In the guideline of the European Society of Anaesthesiology for postoperative delirium, cardiovascular disease is described as a risk factor. It is also reported that comorbidities and a high degree of American Society of Anaesthesiologists (ASA) Physical Status Classification System pose a significant risk for POD [12]. Preoperative anaemia, as another surrogate marker for morbidity, is declared as a risk factor for POD as well as a predictor for a longer stay in hospital and in Intensive care unit (ICU) [12–16]. The combination of advanced age and comorbidities is often found in patients undergoing invasive and major cardiac surgery.

Many studies describe the increased risk of POD associated with cardiac surgery as 9–73% on average. This variability depends on several factors, such as the characteristics of the patients, the length of stay (LOS) in ICU and the delirium testing modalities. The difference between retrospective data collection using ICD codes and prospective daily testing for delirium by trained personnel is substantial [17–23]. Therefore, the subgroup analysis is focused on the comparison of the frequency of positively tested delirium compared to coded ICD (ICD-10-German Modification) diagnosis delirium in the same patient group.

The occurrence of postoperative delirium affects the workload of nursing staff and has a negative impact on patient outcomes. POD is associated with prolonged ICU and hospital stay, increased mortality and costs [9, 20, 22, 24–31]. To further investigate the influence of POD on LOS, this subgroup analysis includes possible surrogate parameters for morbidity that may influence both POD and LOS. Prolonged LOS in ICU poses a big burden on the limited resources of intensive care beds [32, 33]. Based on these results, the S3 guideline "Analgesia, Sedation and Delirium Management in Critical Care" calls for risk screening and preventive intervention and treatment of POD to reduce the incidence of delirium [34].

Therefore, the aim of this subgroup analysis was to measure the relationship of coded delirium diagnosis in comparison to the actual incidence of tested delirium in patients undergoing cardiac surgery. Furthermore, this study explored the severity of the diseases and analysed the impact of delirium on the length of ICU and hospital stay.

## Methods

### Study population

This prospective monocentre observational trial included 1098 patients from different surgical disciplines of the University Hospital Bonn. From September 2018 to October 2019, all patients, older than 60 years and with planned operations of at least 60 min duration, were considered eligible for the study. This study was conducted under the title: PRe-Operative Prediction of postoperative DElirium by appropriate SCreening (PROPDESC) and was registered in the German Registry for Clinical Studies under the following number DRKS00015715 [35]. The subgroup analysed here consists of all patients with cardiac surgery included in PROPDESC. The enrolled cohort of 308 patients consisted mainly of coronary artery bypass surgery (CABG), valve replacement or repair, or combined CABG with valve replacement or repair. Exclusion criteria included emergency procedures, language barriers or missing compliance with the study protocol. The present study complied within the principles of the declaration of Helsinki and was approved by the local institutional Ethics Committee at the Medical Faculty of the Rheinische Friedrich-Wilhelms-University of Bonn. Written informed consent was obtained from each patient.

### Data collection

For each enrolled patient, 50 variables were collected. In this subgroup analyses, preoperative risk stratification such as ASA Physical Status Classification System, age, sex, number of medications, haemoglobin and the type of surgery were applied. Postoperative clinical variables were recorded including length of the intensive care unit stay (ICU-LOS) and LOS in the hospital. After discharge from the hospital, billing-relevant data such as the number of ICD codes and the severity of inpatient treatment were evaluated for each patient in the form of the German Patient Clinical Complexity Level (PCCL). In the German Diagnosis Related Group (G-DRG) classification, complications and/or comorbidities (CC) are mapped using the patient-related total severity code (PCCL). The PCCL is calculated from the cumulative severities of complications and/or comorbidities (CCL) of a patient's individual.

The data for the external comparison with regard to ICD-10-GM coding and the information on PCCL and LOS was taken from the Institut für das Entgeltsystem im Krankenhaus gGmbH (InEK) browser database 2019 [36]. The classification for postoperative delirium is listed in the ICD-10-GM Catalogue under Chapter V with the class title "Mental and Behavioural Disorders" under category F05.- with the designation "delirious, not caused by alcohol or other psychotropic substances". The number of positive delirium results assessed by study personnel were compared to those coded by the hospital staff (ICD codes F05.0 and F05.8). To classify the individual cardiac surgery procedures, the German Operation and Procedure Code (OPS) classification 2019 was used.

### Delirium assessment

Delirium assessments were conducted every morning by trained doctoral students on each of the first 5 days after surgery, respectively, on the first 5 days´ post-sedation. For this purpose, several standardized tests were used. To avoid missing delirium diagnosis in the context of spot examinations, the Delirium Observation Scale (DOS) was additionally applied by interviewing the nursing staff to assess the previous 24 h. Regarding the 5-day visit period, we used different test procedures for the intensive care and normal ward. Confusion Assessment Method for ICU (CAM-ICU) was used for intensive care patients. The Confusion Assessment Method (CAM) and the 4 'A's Test (4AT) were conducted in patients on the normal ward. The endpoint of a positive delirium diagnosis was considered to be fulfilled if one of the applied assessment methods detected POD on at least one of the 5 days. The aim of the overall PROPDESC study was to establish a sensitive risk score for postoperative delirium, thus different testing procedures were used in parallel to avoid missing any delirium abnormalities in the study cohort. Based on this subgroup analysis, the primary endpoint was maintained based on a positive test result from the various assessment tools. In accordance with good clinical practice, doctoral students were trained and monitored in the performance of each test at the beginning of the study. Regular quality assurance meetings were held throughout the study [35].

### Data analysis

Statistical analysis was performed using the statistical programming environment R. Continuous and ordinal variables are presented with mean and ± standard deviation (sd). Nominal variables are displayed as numbers and percentages. Furthermore, the comparison between the delirium tested by trained study personnel and the coded delirium at the University Hospital Bonn and the average in Germany is presented by means of percentages. The grouping of individual procedures was performed on the basis of the billed diagnosis related groups (DRG). The same procedure was used to assess the LOS and the severity of treatment with PCCL.

Differences between the two groups (POD versus no POD) regarding preoperative factors were analysed using the non-parametric Mann–Whitney test for continuous variables. For categorical variables, Fisher’s exact test was computed to check for independence.

To assess the independent effect of POD on LOS in ICU or in hospital, multi-variable linear regression analysis was performed to adjust for potential preoperative confounders. The LOS outcomes were log-transformed to ensure approximate normality of residuals. POD was entered as binary explanatory variable, while adjusting for preoperative surrogate parameters for morbidity (age, number of medication, ASA, preoperative haemoglobin value). To ensure interpretability, the coefficients of POD were re-transformed and are presented in percent increase (compared to non-POD) with corresponding 95% confidence intervals reflecting adjusted relative effects of POD on LOS.

## Results

**Fig. 1:**
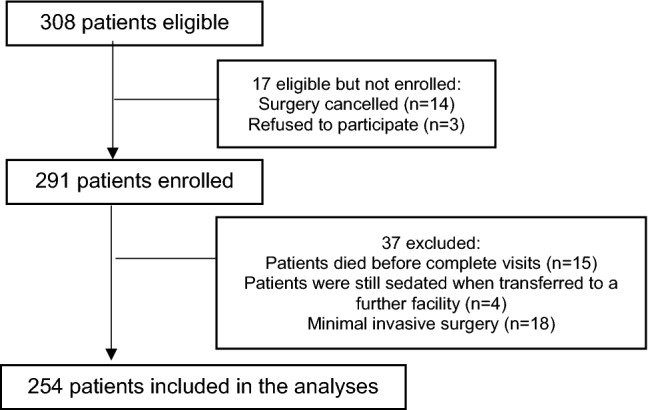
Flowchart of subgroup patient selection

The subgroup included 308 patients, 14 (4.5%) patients were not operated, 15 (4.9%) patients died and 4 (1.3%) patients were still sedated when transferred to a further facility. 18 (5.8%) of the included patients received pacemaker or minimal invasive surgery and were removed from the analysed patient group based on the lack of complexity of the procedure. Three (1.0%) patients have withdrawn their consent and are, therefore, considered to be study dropout (Fig. 1). Thus, 254 patients with a mean age of 70.5 (± 6.4) years were included in the analyses. The gender distribution was 72 (28.4%) women and 182 (71.7%) men. We divided these patients into two groups based on the presence or absence of tested delirium: the POD group (*n* = 127, 50.0%) and the non-POD group (*n* = 127, 50.0%). For the evaluation on the basis of the billed DRG, one case is not included, since this case was billed with the previously performed pacemaker operation despite the heart valve operation.

### Procedural coding of POD

The ICD code F05.0 "Delirium without dementia" was coded 38 (15.0%) times in the included patient group of the University Hospital Bonn and the diagnosis F05.8 "Other form of delirium" was coded 15 (5.9%) times. Among the F05.0 positively coded patients, 33 (86.8%) were tested positive by the study personnel in the specified assessment window and 14 (93.3%) of the F05.8 coded patients have also been tested positive. The percentage of positive POD patients in PROPDESC is consistently higher than the coded diagnoses in the University Hospital Bonn and the German average (Table 1). The tested delirium incidence ranges from an average of 35.5% up to 100%. In our trial, we found a rate of positive tested delirium for patients with heart valve surgeries of 58.5%, whereas the InEK data of coded ICD diagnoses for delirium was in total 14.3% in this group of patients. For CABG patients in our trial the incidence of delirium was 35.6%, whereas the InEK shows an average percentage of 18.7%. In addition, we found that the percentage frequency of coded delirium diagnoses at the University Hospital Bonn (CABG 11.5%; heart valve surgery 18.1%) was considerably lower than that of the same patients who were tested positive by study personnel (CABG 35.6%; heart valve surgery 58.5%). In addition to the ICU complex treatments, the combination procedures involving CABG and heart valve surgery (71.4%) showed the highest tested delirium incidence.

**Table 1:** Distribution of ICD codes and positive POD test results

		Total	POD group		POD group
			F05.0	F05.8	PROPDESC
		n	% (n)	% (n)	% (n)
Heart valve surgery					
	University hospital	94	14.9 (14)	3.2 (3)	58.5 (55)
	InEK database	5,929	2.8	11.5	
CABG					
	University hospital	87	8.1 (7)	3.5 (3)	35.6 (31)
	InEK database	6,870	3.8	14.9	
Complex intervention					
	University hospital	7	0.0	14.3 (1)	71.4 (5)
	InEK database	1,575	1.2	8.9	
ICU complex treatment					
	University hospital	10	60.0 (6)	20.0 (2)	90.0 (9)
	InEK database	1,730	9.3	23.2	
Ventilation > 24 h					
	University hospital	31	12.9 (4)	6.5 (2)	35.5 (11)
	InEK database	1,366	5.6	13.0	
Ventilation > 95 h					
	University hospital	13	23.1 (3)	0.0	38.5 (5)
	InEK database	4,075	7.3	16.0	
Ventilation > 249 h					
	University hospital	4	50.0 (2)	0.0	75.0 (3)
	InEK database	2,731	8.8	14.6	
Ventilation > 499 h					
	University hospital	3	33.3 (1)	33.3 (1)	100.0 (3)
	InEK database	180	13.9	15.0	
Ventilation > 1799 h					
	University hospital	4	25.0 (1)	50.0 (2)	100.0 (4)
	InEK database	83	18.8	22.1	

This table shows the relative frequency of coded delirium diagnoses (ICD F05.0 and F05.8) at the Bonn University Hospital and in the InEK database, as well as the relative frequency of positively tested delirium patients. For ease of comparison, the individual billing codes (DRGs) are grouped under the terms of the main interventions. The following DRGs are summarized under the respective main interventions: Heart valve surgery included DRGs: F03A-F03C, F03E-F03F; CABG included DRGs: F05Z, F06A-F06E; Complex intervention included DRGs: F07B-F07C; ICU complex treatment included DRGs: F36A-F36C; Ventilation > 24 h included DRG: F43B; Ventilation duration > 95 h included DRGs: A13A, A13D-A13E; Ventilation duration > 249 h included DRGs: A11A-A11B, A11E; Ventilation duration > 499 h included DRG: A09A; Ventilation > 1799 h included DRGs: A06A-A06B

POD postoperative delirium, CABG coronary artery bypass grafting, ICU intensive care unit, InEK Institut für das Entgeltsystem im Krankenhaus gGmbH

### Comparison of comorbidity

The average severity of disease, expressed in PCCL, was not predominantly higher in university hospital patients than in the patients in the InEK comparison (Table 2). This is explained by the fact that most cardiac surgeries are performed at university hospitals and therefore the severity of the InEK patients is similar to the severity of the patients examined here. Within the entire subgroup, 188 (74.3%) patients were billed on the basis of cardiac surgery and 65 (25.7%) on the basis of the more complex intensive care treatment after the cardiac surgery. The presence of delirium showed statistically significant differences in the preoperatively determined haemoglobin value (no delirium: 14.0 ± 1.6 g/dl; delirium: 13.4 ± 1.8 g/dl; *p* < 0.009), the number of ICD codes (no delirium: 13.5 ± 5.3; delirium 18.5 ± 10.3; *p* < 0.001) and the level of PCCL (no delirium: 2.6 ± 1.4; delirium 3.4 ± 1.5; *p* < 0.001) after discharge from hospital (Table 3). The ASA classification, number of different medication taken before surgery and age of the patients did not differ significantly between the delirious and non-delirious patients. The mean age was 70.9 (± 6.4) years for the delirious patients and 70 (± 6.3) years for the non-delirious patients. Based on their underlying cardiac disease, 77% (n = 195) of patients were grouped with ASA 3 (Table 3).

**Table 2:** List of severity levels by *Patient Clinical Complexity Level (*PCCL) and average length of stay (LOS)

		Total	LOS in hospital (days)	PCCL 0	PCCL 1	PCCL 2	PCCL 3	PCCL 4	PCCL 5	PCCL 6
		N	mean	% (n)	% (n)	% (n)	% (n)	% (n)	% (n)	% (n)
Heart valve surgery										
	University hospital	94	18.6	20.2 (19)	1.1 (1)	7.5 (7)	35.1 (33)	26.6 (25)	9.6 (9)	0.0 (0)
	InEK database	5,929	14.5	19.2	6.5	14.2	28.4	23.8	7.8	0.2
CABG										
	University hospital	87	15.1	10.3 (9)	10.3 (9)	21.8 (19)	31.0 (27)	18.4 (16)	8.1 (7)	0.0 (0)
	InEK database	6,870	19.0	8.2	6.6	11.7	21.2	34.8	16.3	1.3
Complex intervention										
	University hospital	7	13.6	14.3 (1)	0.0 (0)	0.0 (0)	28.6 (2)	42.9 (3)	14.3 (1)	0.0 (0)
	InEK database	1,575	13.5	14.7	3.9	9.4	28.3	28.4	14.4	1.0
ICU complex treatment										
	University hospital	10	30.8	0.0 (0)	0.0 (0)	0.0 (0)	0.0 (0)	40.0 (4)	50.0 (5)	10.0 (1)
	InEK database	1,730	29.6	0.5	1.0	1.9	13.8	33.1	41.8	8.0
Ventilation > 24 h										
	University hospital	31	15.8	0.0 (0)	3.2 (1)	16.1 (5)	54.8 (17)	25.8 (8)	0.0 (0)	0.0 (0)
	InEK database	1,366	17.6	2.3	3.3	10.4	35.5	45.6	2.9	0.0
Ventilation > 95 h										
	University hospital	13	23.5	0.0 (0)	7.7 (1)	7.7 (1)	53.9 (7)	23.1 (3)	7.7 (1)	0.0 (0)
	InEK database	4,075	23.1	3.7	3.1	6.3	28.4	42.0	16.2	0.2
Ventilation > 249 h										
	University hospital	4	32.3	0.0 (0)	0.0 (0)	25.0 (1)	0.0 (0)	50.0 (2)	25.0 (1)	0.0 (0)
	InEK database	2,731	34.3	2.7	2.7	7.1	27.8	37.8	20.5	1.4
Ventilation > 499 h										
	University hospital	3	93.7	0.0 (0)	0.0 (0)	0.0 (0)	0.0 (0)	0.0 (0)	66.7 (2)	33.3 (1)
	InEK database	180	58.5	0.0	0.0	0.6	2.2	25.0	61.1	11.1
Ventilation > 1799 h										
	University hospital	4	132.8	0.0 (0)	0.0 (0)	0.0 (0)	0.0 (0)	0.0 (0)	25.0 (1)	75.0 (3)
	InEK database	83	111.9	0.0	0.0	0.0	3.5	7.6	39.5	49.3

This table shows the comparison of the Patient Clinical Complexity Level (PCCL) of the tested patients of the University Hospital Bonn and the data of the InEK. For ease of comparison, the individual billing codes (DRGs) are grouped under the terms of the main interventions. The following DRGs are summarized under the respective main interventions: Heart valve surgery included DRGs: F03A-F03C, F03E-F03F; CABG included DRGs: F05Z, F06A-F06E; Complex intervention included DRGs: F07B-F07C; ICU complex treatment included DRGs: F36A-F36C; Ventilation > 24 h included DRG: F43B; Ventilation duration > 95 h included DRGs: A13A, A13D-A13E; Ventilation duration > 249 h included DRGs: A11A-A11B, A11E; Ventilation duration > 499 h included DRG: A09A; Ventilation > 1799 h included DRGs: A06A-A06B

PCCL German Patient Clinical Complexity Level, LOS length of stay, InEK Institut für das Entgeltsystem im Krankenhaus gGmbH, CABG coronary artery bypass grafting, ICU intensive care unit

**Table 3:** Perioperative risk factors for POD

	Total	POD group	Non-POD group	*p* value
No. (%)	254	127 (50.0)	127 (50.0)	-
Age (years)	70.5 ± 6.4	70.9 ± 6.4	70.0 ± 6.3	0.229
No. of coded ICD	16.0 ± 8.6	18.5 ± 10.3	13.5 ± 5.3	< 0.001
No. of medication	6.1 ± 2.9	6.3 ± 3.0	5.9 ± 2.8	0.196
Haemoglobin (g/dl)	13.7 ± 1.7	13.4 ± 1.8	14.0 ± 1.6	0.009
Level of PCCL	3.0 ± 1.5	3.4 ± 1.5	2.6 ± 1.4	< 0.001
PCCL level 0		7.0 (9)	15.9 (20)	
PCCL level 1		3.9 (5)	5.6 (7)	
PCCL level 2		11.7 (15)	14.3 (18)	
PCCL level 3		26.8 (34)	40.9 (52)	
PCCL level 4		28.9 (37)	19.8 (25)	
PCCL level 5		17.2 (22)	4.0 (5)	
PCCL level 6		3.9 (5)	0.0 (0)	
Level of ASA	3.2 ± 0.5	3.2 ± 0.5	3.2 ± 0.5	0.638
ASA level 1		0.8 (1)	0.0 (0)	
ASA level 2		1.6 (2)	4.0 (5)	
ASA level 3		77.2 (98)	76.4 (97)	
ASA level 4		20.3 (26)	19.8 (25)	
LOS in hospital (days)	20.6 ± 20.0	26.5 ± 26.1	14.6 ± 6.7	< 0.001
LOS ICU (hours)	114.8 ± 266.8	165.0 ± 362.7	64.5 ± 79.4	< 0.001

POD postoperative delirium, PCCL German Patient Clinical Complexity Level, ASA American Society of Anaesthesiologists, LOS length of stay, ICU intensive care unit

Data are expressed as mean ± standard deviation. The frequencies of the individual levels of PCCL and ASA are shown in percent and (= n)

### Relationship between delirium and LOS

Table 2 compares the average LOS of patients of the PROPDESC study patients at the University Hospital Bonn with the average LOS of patients in the InEK database. Patients at the University Hospital had different LOS (valve surgery 18.6 days; CABG 15.1 days) compared to the mean value of the InEK population (valve surgery 14.5 days; CABG 19.0 days). Patients manifesting delirium had a significantly longer LOS in hospital (no delirium: 14.6 ± 6.7 days; delirium 26.5 ± 26.1 days; *p* < 0.001) (Table 3). Furthermore, the study results display that patients with a POD are hospitalized on average 12 days longer (Table 3). The study results confirm that the LOS in hospital is nearly twice as long in patients with POD after cardiac surgery (26.5 ± 26.1 days) compared to the average LOS of this patient group (14.6 ± 6.7 days). The results of the linear regression model support this statement (Table 4). They showed that patients with POD have a 48% (95% CI 31–67%) increase in LOS in hospital even when adjusting for potential confounders.

**Table 4:** POD as an independent predictor for LOS in the ICU and in hospital: effects were adjusted for preoperative risk factors via a multi-variable linear regression model and are presented as increase in percent

	POD (adj. effect)	95% CI	*p* value
LOS in ICU (hours)	+ 48%	+ 31% to + 67%	< 0.001
LOS in hospital (days)	+ 64%	+ 27% to + 110%	< 0.001

POD postoperative delirium, CI Confidence Interval, ICU intensive care unit, LOS length of stay

POD effect adjusted for preoperative surrogate parameters for morbidity (age, number of medication, ASA, preoperative haemoglobin value)

In addition to this, the study results demonstrate that patients with delirium had a significantly longer ICU LOS (no delirium: 64.5 ± 79.4 h; delirium 165.0 ± 362.7 h; *p* < 0.001) (Table 3). In total, the delirious study patients had a 2.5 times longer intensive care stay than the group of patients without delirium. The average time difference was 100 h and was caused by the fact that study patients with delirium stayed 4.2 days longer in ICU. The results of the linear regression confirm delirium as an independent predictor of LOS in ICU (Table 4). Following our model, patients with POD have a 64% (95% CI: 27–110%) increase in LOS in ICU independently from their preoperative risk factors.

## Discussion

POD is a common complication of elderly patients after cardiac surgery and has a high impact on LOS in ICU and hospital. Furthermore, the secondary diagnosis of POD is clearly underdiagnosed, demonstrating the extent to which this secondary diagnosis is underestimated. The incidence in the present study was 50.0% and thus in between the 9% and 73% stated in the literature [17–23]. Explanations for this variability in the literature could be a different extent and different instruments of studies to assess POD [11]. While PROPDESC used several tools (two for ICU and three on normal ward) other studies evaluated POD with one tool or used the retrospective analysis of ICD codes. In this study, we compared the number of positive tested delirious patients (from 35.5 to 100%) with the coded delirious diagnosis (ICD F05.0 and F05.8) in the University Hospital Bonn and the German-wide average (from 10.3 to 40.9%). We found that the percentage frequency of reported delirium diagnoses in the considered group of patients was significantly lower than as tested positive by study personnel. The difference was 40.4% for heart valve surgery and 24.1% for CABG. There are several explanations for this significant difference. Prior work has described a range up to 80% of the hypoactive subtype of delirium [6, 10, 20, 23, 37, 38]. These results suggest that the form of hypoactive POD often remains undetected by hospital staff and is, therefore, not so present in the reported ICDs. Furthermore, this could also lead to the assumption that there is no standardized delirium testing, as pointed-out by various studies and guidelines [12, 39–45]. It should also be noted that in the German DRG system, the share of material costs for heart valve surgery and CABG, accounts for more than a quarter of total costs (heart valve surgery F03A-F03F 30–37% material costs; CABG F05Z, F06A-F06E 23–30% material costs) [36]. Considering the high material costs, the coding of delirium does not result in a relevant surcharge and might be, therefore, neglected as a complication and comorbidity. This leads to the conclusion that from a medico-economic perspective there is no incentive to diagnose POD. However, various examples can be found in the existing literature that the nursing effort in combination with a POD increases significantly and thus, the cost-relevant effort as well [9, 20, 46].

Prior work has documented that a high number of comorbidities, severe diseases and advanced age occur more frequently among the delirious patients [12]. Our data confirm the results of previous studies that comorbidities have an influence on the development of POD which is shown by the significantly higher number of ICD codes and PCCL of delirious patients [2, 12, 20, 38]. However, we could not find a significant difference between the POD and the non-POD groups in terms of age, number of preoperative medication and ASA classification preoperatively. So far, only few studies have dealt with the hypothesis whether patients have a more complex and longer course of inpatient treatment due to delirium, or whether the present morbidity is the main reason for this. One study confirmed that the prolonged intensive care stay of cardiac surgery patients is based on the complication of POD and not on the pre-existing morbidity [20]. However, POD is very often recognized as an effect on ICU LOS and length of hospital stay [20, 47–51]. In this study, we were able to show that POD is independently associated with an increased LOS in ICU and in hospital among patients undergoing cardiac surgery and, also an extended length of hospital stay compared to the German average. Based on the PCCL comparison between the PROPDESC patients and the German average, the argument that the study population is sicker and, therefore, has a longer stay is not supported by the results of our analysis. On the contrary, we were able to confirm via multiple linear regression that POD has an independent effect on LOS after adjusting for preoperative surrogate parameters for morbidity.

Although the results of this subgroup study analysis demonstrate causality only for surrogate parameters, they underline the importance of detection of POD in elderly cardiac surgery patients. Delirium poses additional work on the nursing staff and prolongs the duration of the ICU stay by an average of 4.2 days. If German hospitals would introduce standardized preoperative risk screening and prevention programmes to increase the awareness of a possible POD, the incidence of delirious patients might be reduced [52–56]. If standardized screening with containment prevention and therapy of POD could reduce the LOS in ICU, this would have a considerable impact on the limited capacities of German intensive care units. Among the 254 patients included in this study over the period of 1 year, approximately 50% were delirious after their surgery. A reduction of the LOS of this patient group by one day (from an average of 7 to 6 days) would result in the free capacity of an intensive care bed for 127 days per year. According to the University Hospital Bonn's quality report, 720 CABG and heart valve surgeries were performed in 2018. If an extrapolated 50% of the patients in the total population had shown delirium, this would have resulted in 360 patients. By reducing the LOS on ICU by only 1 day, the capacity of one bed in a 12-bed ICU would be available for about 1 year (360 days) for additional patients.

Based on the results of this study POD has an impact on LOS in ICU and is rarely diagnosed in clinical routine. If the delirium diagnosis does not have a relevant influence on the billing amount, the reduction of the incidence of delirium should be focussed on medical-economic aspects to improve the capacity utilization of the bottleneck in ICUs.

## Study limitation

This study has several limitations. One limitation is the small sample size, related to the character of the subgroup analyses. In connection with the regression analysis, there might be unknown confounding factors for which we were not able to adjust for. These factors could additionally influence both POD and the LOS. Furthermore, the delirious PROPDESC patients were only based on the result of a positive test result of the study staff and has no delirium diagnosis by a psychologist. The comparison with the nationwide ICD diagnoses of POD and other data from the InEK browser database is based on data from 2019, but the patients of this subgroup were enrolled during 2018 and 2019. Based on the coding guidelines, only ICDs with associated inpatient treatment costs are coded and, therefore, do not represent the total comorbidities of patients. In addition, the summarized Tables with the DRG overview do not clearly show which interventions the intensive complex treatments are based on.

## Conclusion

Postoperative delirium is associated with a significantly increased LOS in hospital as well as ICU. The frequency of ICD coding of POD in the subgroup analysis as well as in the internationally available accounting data is considerably lower than the tested incidence of POD. Based on the underlying billing system, there is no financial incentive for ICD coding of POD in cardiac surgery patients, so this could be a possible reason for the low coding rate of this secondary diagnosis. Future research should evaluate the introduction of standardized, fast and simple preoperative risk screening followed by prevention programmes to reduce the incidence of delirium and its impact on LOS.

## Data Availability

The data sets generated and/or analysed during the study are available on request from the corresponding author.

## Code availability

The R code used for the analysis is available on request from the corresponding author.
